# Congenital Cheek Fistula: A Rare Case of Developmental Abnormality in the Buccal Area

**DOI:** 10.7759/cureus.15657

**Published:** 2021-06-15

**Authors:** Daiki Kitano, Shunsuke Sakakibara, Yasuhisa Ishida, Tadashi Nomura, Hiroto Terashi

**Affiliations:** 1 Department of Plastic Surgery, Kobe University Graduate School of Medicine, Kobe, JPN

**Keywords:** congenital cheek fistula, transverse facial cleft, orbicularis oris muscle, abnormality, buccal area, rare

## Abstract

A congenital cheek fistula is a rare malformation in the buccal area. Here, we report the case of a congenital cheek fistula in a 50-year-old woman who visited our clinic with complaints of swelling and pain in her left cheek. Physical examination revealed a small hole in the left corner of the mouth present since birth. She had no other congenital malformations in the maxillofacial region such as an accessory ear and cleft lip. Manual compression of the cheek mass induced serous discharge from the hole. Magnetic resonance imaging (MRI) showed a cystic lesion in the left cheek and a fistula within the orbicularis oris muscle that opened into the small hole. After immediate incision and drainage of the cyst, both the cyst and fistula were surgically resected. The cystic lesion was completely delineated from the boundary of the parotid gland. The orbicularis oris muscle was partially incised to remove the fistula and the surrounding scar tissue. Histopathological examination of the resected specimen revealed a cavity consisting of epithelium inside the fistula. The postoperative course was insignificant. No recurrence of the cyst was observed six months postoperatively. The operative and pathological findings demonstrated that the ectoderm-derived epithelial tissue was enclosed by the mesoderm-derived muscle tissue. The mixture of different germ layer-derived tissues suggested that the fistula was a type of congenital transverse facial cleft induced by malfusion of the mandibular and maxillary prominences during embryonic development. The differential diagnoses of the congenital cheek fistula included orocutaneous fistulas and salivary fistulas. MRI was useful in delineating the border between the lesion and the surrounding tissue.

## Introduction

A congenital cheek fistula is a rare condition that presents as a hole in the buccal area. Because congenital cheek fistula is classified as Tessier number 7, it is assumed to be caused by incomplete malfusion of the maxillary and mandibular prominences during early development [1,2]. Patients with congenital cheek fistulas rarely seek medical attention because the majority of cases are asymptomatic. There is a paucity of literature regarding the details of the congenital cheek fistula, including diagnostic studies, differential diagnoses, and pathological findings. Here, we report a case of a congenital cheek fistula in a middle-aged woman.

## Case presentation

**Figure 1:**
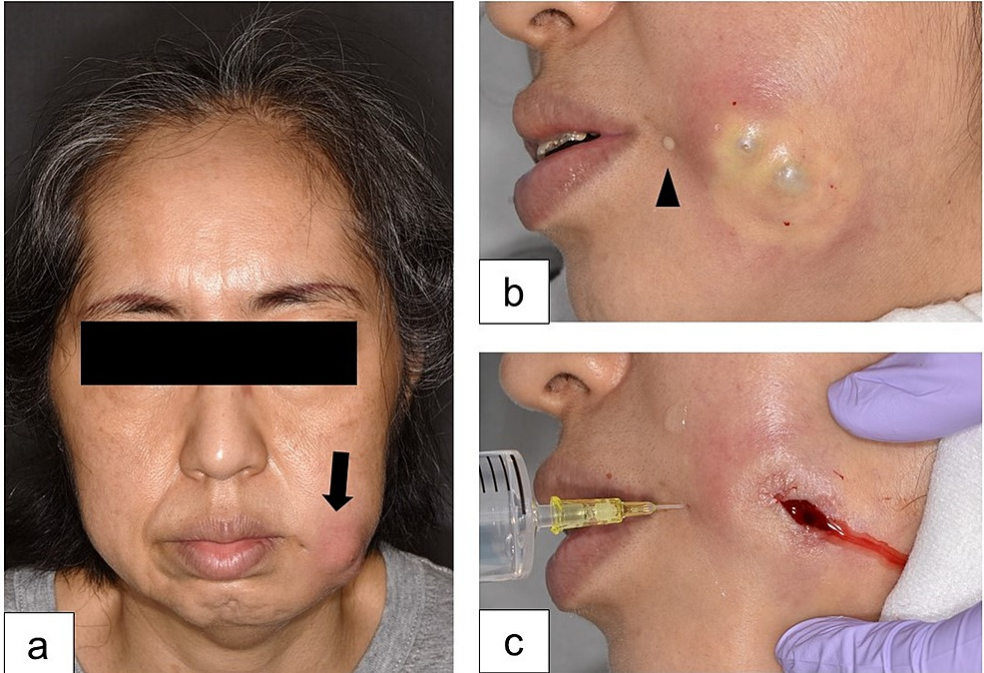
Clinical observations during the patient’s first visit. (a) An erythematous lump (arrow) in the left buccal area and a small hole at the corner of the mouth are visible. (b) Manual compression of the buccal lump yielded white discharge from the small hole (arrowhead). (c) Saline irrigation and drainage after incision of the buccal lump suggested a subcutaneous connection between the hole and the lump.

A 50-year-old woman was referred to our hospital for a left cheek lump associated with severe pain (Figure 1a). Her medical history revealed gallbladder resection and mood disorders. The patient did not report any problems with her growth, and she had no congenital malformations in the head and neck region. No one in her family had developed similar symptoms before.

Physical examination revealed that she had a small hole in the left cheek close to the angle of the mouth. She had a fistula since birth and sometimes experienced serous secretion that was not enhanced on eating. Manual compression of the cheek lump prompted serous secretion from the hole (Figure 1b).

**Figure 2:**
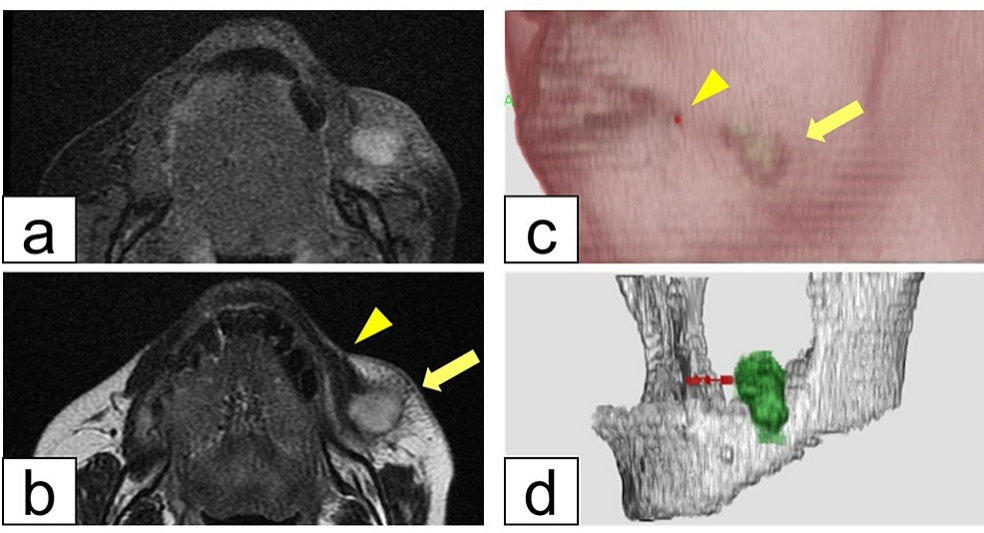
MRI findings. (a) STIR image suggests severe inflammation of the cystic lesion. (b) T2-weighted image reveals a subcutaneous cystic lesion (arrow) and a fistula within the muscle tissue (arrowhead). (c and d) Left-sided view of the three-dimensional MRI model. Soft tissue spectral subtraction images demonstrate communication between the opening of the fistula (arrowhead) and the cystic lesion (arrow). MRI: magnetic resonance imaging; STIR: short T1 inversion recovery

Magnetic resonance imaging (MRI) revealed a cystic lesion in the left cheek with a high-signal area on diffusion-weighted imaging (Figure 2a). The cystic lesion was isolated from the parotid gland on T2-weighted imaging (Figure 2b). Three-dimensional T2-weighted imaging demonstrated a subcutaneous connection between the cystic lesion and the small hole (Figures 2c, 2d). MRI findings implied that the lump was an inflamed subcutaneous abscess.

An immediate skin incision of the lump yielded a white purulent discharge that was compatible with the MRI findings indicating that the perioral hole communicated subcutaneously with the cyst (Figure 1c). Culture testing of the discharge indicated the presence of *Staphylococcus aureus* and *Prevotella bivia*. The connection between the perioral hole and the oral cavity was not evident. The wound was washed with normal saline and povidone-iodine ointment was applied daily.

**Figure 3:**
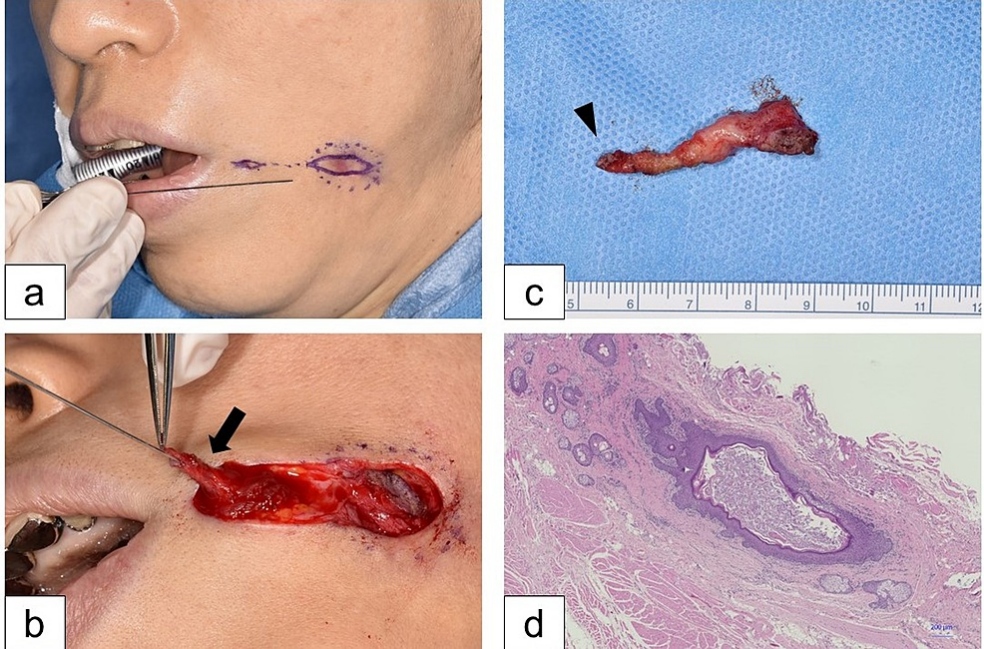
Operative findings. (a) The fistula and cystic lesion, including the surrounding skin and scar tissue, are resected. (b) The fistula is enclosed within the orbicularis oris muscle tissue (arrow). (c) The resected specimen. The arrowhead indicates the opening of the fistula. (d) Hematoxylin-eosin staining (magnification ×10) of the specimen shows that the inner lumen of the fistula is composed of stratified squamous epithelium, which is surrounded by a layer of interstitial tissue. Muscle fibers are observed in the outer part of the resected specimen.

After the resolution of the inflammation, resection of the cystic lesion was performed under general anesthesia (Figure 3a). Crystal violet (Pyoktanin BlueTM; Fujifilm, Tokyo, Japan) was injected into the hole, staining the inside of the cystic lesion. The cystic lesion was resected by delineating the border between the lesion and the surrounding tissue. The orbicularis oris muscle was partially incised to resect the fistula within the muscle (Figures 3b, 3c). Pathological examination of the resected specimen confirmed a cavity lined with epithelial tissue (Figure 3d). The muscle tissue was repaired with 4-0 Vicryl sutures and the wound was primarily closed.

**Figure 4:**
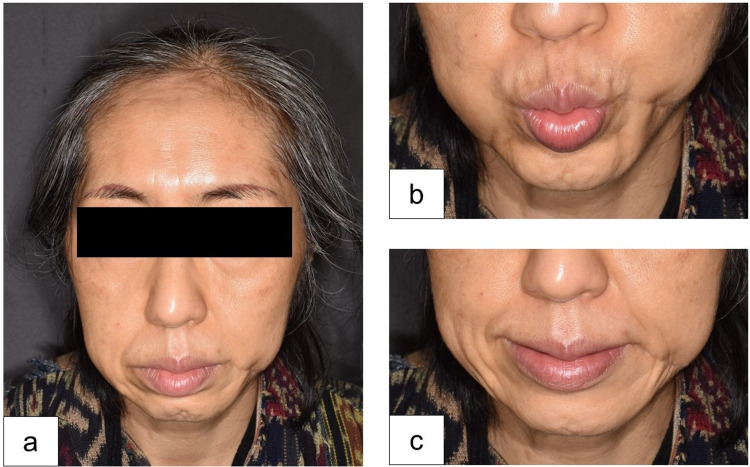
Clinical observations during the six-month follow-up visit. (a) The mature operative scar. (b and c) No motor disturbance observed at the corner of the mouth.

The patient’s postoperative course was uneventful. No cyst recurrence or infection occurred during the postoperative six months (Figure 4a). Moreover, the patient did not develop facial nerve palsy (Figures 4b, 4c).

## Discussion

The facial cleft is a congenital morphological abnormality of the bone and soft tissue of the maxillofacial area. Cleft lip and palate are the most common types of facial cleft, with other atypical facial clefts being less common. The transverse facial cleft is characterized by a cleft that extends from the angle of the mouth to the ear, thus corresponding to Tessier classification number 7 [1]. There are two major hypotheses regarding the cause and development of a facial cleft: malfusion of the facial prominences during the embryonic stage, or the failure of the mesodermal tissue to achieve invasion. Malfusion of the maxillary and mandibular prominences, which are derived from the first branchial arch, is considered to be the leading cause of the transverse facial cleft. The severity of the cleft depends on the degree of malfusion. Incomplete malfusion of these prominences results in a fistula.

The existing literature regarding the congenital cheek fistula is limited; however, a periauricular fistula is one of the most common congenital malformations in the craniofacial area. Nakano et al. reported three neonatal cases of cheek fistulas classified as Tessier number 7 [2] and suggested that the congenital cheek fistulas were incomplete transverse facial clefts. The fistula in our patient was also classified as Tessier number 7, which was consistent with their suggestion. Additionally, the fistula contained ectodermal tissue and was covered with the orbicularis oris muscle, originating from the mesoderm [3]. These findings increased the possibility that the fistula was induced by incomplete malfusion of the maxillary and mandibular prominences. As a result, the epidermal component remained within the muscle tissue at birth. Although this congenital malformation was asymptomatic, occlusion of the fistula triggered inflammation caused by the accumulated contents.

One of the differential diagnoses of congenital cheek fistula includes orocutaneous fistula, which represents a communication between the oral cavity and the outer skin surface. Patients with an orocutaneous fistula and cleft lip are classified as having Van der Woude syndrome [4,5]. In our case, no communication between the fistula and the oral cavity was evident. The patient denied any history of facial cleft, including cleft lip or palate.

Another possible differential diagnosis is a congenital salivary fistula of the parotid gland. The parotid gland opens into the oral cavity through the Stensen duct; however, various factors, such as trauma or surgery, cause aberration of the duct opening into the skin, resulting in a parotid cutaneous fistula [6]. Congenital parotid cutaneous fistulas arise from the abnormal opening of the Stensen duct [7], or the accessory parotid gland, which exists separately from the main parotid gland [8-10]. Patients with a parotid cutaneous fistula often report secretion from the fistula while eating. In our case, the communication between the fistula and the parotid gland was not clear. Moreover, the patient confirmed intermittent drainage from the fistula but denied its acceleration while eating.

Fluoroscopy with a contrast agent is a reliable diagnostic imaging technique for fistulas [6]. Computed tomography with fistulography offers a three-dimensional view of the complexity of the fistula [9]. However, radiation exposure and the injection of contrast medium within the fistula are major concerns associated with fistulography. Ultrasonography is another diagnostic imaging technique for fistulas [11]. However, patients with severe infection report pain with the use of the probe during the compressive procedure performed for the lesion. In our case, we suspected that severe inflammation of the subcutaneous tissue induced a diffuse hyperechoic area, which made it difficult to delineate the border of the lesion.

MRI has been reported to be an alternative scanning tool for fistulas because of its low invasiveness and high-resolution images [12]. Gadodia et al. reported a case of a congenital accessory parotid cutaneous fistula that was diagnosed using MRI and fistulography [13]. They reported that MRI was superior in terms of delineating the boundary between the fistula and the parotid gland; however, the concomitant use of fistulography offered a more accurate image of the fistula. In our case, we selected MRI instead of fistulography because the patient reported severe pain caused by inflammation. Subsequently, preoperative MRI revealed a clear boundary between the lesion and its surrounding tissue.

## Conclusions

We report a rare case of a congenital cheek fistula with inflammation. The operative findings demonstrated that the fistula extended through the orbicularis oris muscle, suggesting that the fistula was a type of transverse facial cleft caused by malfusion of the maxillary and mandibular prominences. MRI clearly showed the boundary between the cystic lesion and the surrounding tissue as well as the course of the fistula.
